# A higher global diet quality score is associated with lower risk of obesity among male university students in Lebanon: a pilot study

**DOI:** 10.3389/fnut.2024.1479448

**Published:** 2024-12-06

**Authors:** Najwa Mourad, Samer Kharroubi, Lara Nasreddine, Nahla Hwalla

**Affiliations:** Department of Nutrition and Food Sciences, Faculty of Agricultural and Food Sciences, American University of Beirut, Beirut, Lebanon

**Keywords:** non-communicable diseases, global diet quality score, obesity, drivers of food consumption, Lebanon

## Abstract

**Objective:**

This pilot study aims to assess the diet quality amongst Lebanese male university students using the Global Diet Quality Score (GDQS), identify its association with obesity, and determine the key drivers of consumption of foods associated with higher NCDs risk.

**Methods:**

A cross-sectional survey was conducted using a convenience sampling approach, comprising 385 male students aged between 18 and 24 years at the American University of Beirut. Dietary data was collected using 24-h recall, where participants detailed all foods and beverages consumed in the past 24 h, including portion sizes. Sociodemographic, anthropometric, lifestyle factors and drivers of food consumption data were also collected. GDQS scores were categorized as high (≥23), moderate (15–23), or low (< 15) indicating low, moderate and high NCD risk, respectively. A multiple logistic regression was applied to assess association of GDQS with sociodemographic and anthropometric variables.

**Results:**

The results showed that the majority of male university students had low (47%) or moderate (47%) GDQS scores, with only a small proportion (4%) showing high scores. Foods contributing to low GDQS scores were determined, with taste emerging as the primary factor influencing food group consumption. Additionally, individuals in health-related majors and higher academic year of study had higher GDQS scores, and those with higher GDQS scores had a lower risk of obesity. The study findings suggest that a high proportion of the study sample are at a higher risk of NCDs given their dietary quality, demonstrated an association between low GDQS scores and obesity risk, and identified education as a predictor of diet quality.

**Conclusion:**

This study calls for larger studies assessing dietary habits and quality amongst Lebanese university students to provide context-specific evidence for the development of targeted interventions aimed at the promotion of healthier eating habits in this population group and curbing the NCDs epidemic in the country.

## 1 Introduction

In both developed and developing nations, non-communicable diseases (NCDs) have emerged as a significant public health concern, constituting a substantial portion of the global health burden. In Lebanon, a small country in the Middle East, high prevalence of NCDs is reported, with hypertension (32.8%), diabetes (26.8%), and cardiovascular disease (16.1%), being the most prevalent (1). NCDs were in fact estimated to account for 91% of all deaths in Lebanon (2), with cardiovascular diseases (CVDs) standing out as the leading cause of death in the country (3). The escalating prevalence of NCDs in Lebanon has been at least partly attributed to the nutrition transition and the adoption of Western lifestyles, influenced by economic, social and commercial factors, which promote unhealthy dietary habits, smoking, physical inactivity and obesity (4). This phenomenon is often more pronounced amongst young adults (18–25 years) in transition from adolescence to adulthood as they become more independent and embark on their higher education/employment journey (5). In fact, this lifecycle stage is typically accompanied by significant changes in food consumption patterns such as eating outside home, breakfast skipping and the adoption of western dietary patterns which contribute to the risk of obesity and excessive weight gain (6). There is no estimate of obesity prevalence amongst 18–25-year adults in Lebanon, but, based on two national studies, the prevalence of obesity was found to increase from 11.9% in 1997 to 20.6% in 2009 amongst adult men aged 20–39 years and from 8.1 to 14.8% amongst women, thus highlighting the extent of the problem. In addition, obesity and health behaviors developed or acquired during this transitional early adulthood stage tends to track into later life, thus impacting the risk of morbidity, mortality and quality of life (5). Amongst these health behaviors, diet quality is increasingly recognized as an important determinant for health and well-being (7).

In this context, dietary metrics serve as invaluable assessment tools to gauge the overall quality of individuals’ dietary intakes by assigning scores to foods and/or nutrient intakes, and sometimes incorporating lifestyle factors, based on their alignment with dietary guidelines. Over time, diverse scoring methods have been employed to evaluate the overall diet quality including the Healthy Eating Index (HEI), Alternative Healthy Eating Index (AHEI), Diet Quality Index (DQI) International and Healthy Diet Indicator (HDI) (8). More recently, and specifically addressing the global surge in obesity and NCDs, the Global Diet Quality Score (GDQS) has been proposed to assess the link between diet quality and NCDs risk (9). The GDQS is a food-based matrix that incorporates both nutrient adequacy and the risk factors associated with non-communicable diseases (NCDs) in its design and scoring methods. It consists of 25 food groups: 16 defined as healthy food groups, 7 as unhealthy food groups, and 2 food groups that are unhealthy when consumed in excessive amounts. The GDQS+ is the total score across the 16 healthy GDQS food groups and the GDQS− is the total score across the 7 unhealthy GDQS food groups and the 2 GDQS food groups that are unhealthy when consumed in excessive amounts (9).

The GDQS has been already tested, validated and utilized in various populations. In Chinese adults, a higher GDQS score was inversely associated with metabolic syndrome and nutrient inadequacy, particularly in younger, female, urban, and higher-educated individuals (10). Among Southern Indian non-pregnant women of reproductive age, GDQS was found to be useful in determining overall nutrient adequacy and certain lipid measures (11). In sub-Saharan African countries, GDQS served as a useful measure for assessing nutrient inadequacy, low MUAC, and anemia in both males and females (12). Similarly, in Mexican women, Castellanos-Gutiérrez et al. demonstrated the GDQS’s effectiveness in evaluating nutrient adequacy and health markers related to chronic disease (13), and in US women, the GDQS showed significant associations with less weight gain, reduced obesity risk, and an inverse correlation with type 2 diabetes risk (14, 15).

Recognizing that young male adults between 18 and 25 years of age represent a neglected age group in comparison with children, adolescents or women in research settings, while also being more difficult to reach (5). This pilot study aims to (1) assess diet quality in a sample of young male adults using the GDQS, (2) investigate the association between GDQS and obesity as an NCD risk factor and (3) identify the factors associated with the consumption of foods associated with NCDs risks. This study would be the first to test the use of the GDQS in the Arab Middle-East context.

## 2 Methods

### 2.1 Study population and design

A cross-sectional survey was conducted on a convenience sample of male students enrolled at the American University of Beirut, aged 18 to 25 years, where students were approached in person at different times and locations on campus, between January and April 2023. Sample size calculations indicated that a minimum of 385 respondents were needed to estimate a conservative prevalence of 50%, with a 95% confidence interval and a 5% margin of error. The sample size was calculated using the World Health Organization (WHO) sample size calculator (16). Inclusion criteria required that participants were Lebanese male students at AUB aged between 18 to 25 years old. Non-Lebanese students and those reporting to be on special or therapeutic diets were excluded.

Data collection was performed by a research nutritionist who underwent extensive training in dietary assessment and interviewing techniques. Data collection was performed on different days of the week, different times of the day and different locations of the university using face-to-face interviews. Each interview lasted for around 20 min. The study was approved by the Institutional Review Board of the American University of Beirut and all subjects provided consent prior to the initiation of data collection. Fieldwork was carried out between January and April 2023.

The multi-component questionnaire used consists of (1) demographic [age (years)] and socioeconomic status (living arrangement, place of residence, major, academic year of study, number of family members living in the family household, number of rooms in the household), (2) self-reported anthropometric measurements [height (cm) and weight (kg)], (3) alcohol consumption (Drinker vs. Non-drinker/past drinker), (4) smoking (Smokers vs. Non Smoker/Past smoker), (5) physical activity frequency (Never; Less than once a week; Once a week; Two or Three times a week; More than three times a week) (16) and physical activity duration (Do not exercise; less than 30 min per day; 30 min; 1–2 h; more than 2 h) (17), (6) dietary assessment: 24-h recall and (7) drivers of consumption of foods.

Dietary assessment was performed through a 24-h dietary recall where participants were asked to recall what they ate and drank the previous day from the time they woke up until the next morning. They were asked to mention the kind of food, quantity, place and time of eating.

The drivers of consumption of foods contributing to negative GDQS score and those contributing to a positive one was conducted. The development of this questionnaire aimed to identify the drivers of consumption for foods that contribute to both positive and negative GDQS scores. The approach was similar to that used in studies examining the barriers and facilitators to food consumption across different populations and among university students (18–22). For the sake of efficiency and to respect respondents’ time, only a select number of food groups from each category were chosen.

The questionnaire includes specific questions targeting the consumption patterns of various food groups, focusing on both barriers and facilitators of consumption. The GDQS− foods included refined grains, sweets and ice cream, sugar-sweetened beverages, and red meat. The GDQS+ positive included fruits, vegetables, low-fat dairy products, deep orange tubers, and whole grains. Each question was designed to allow multiple answers, enabling respondents to select all applicable factors influencing their consumption habits. The questionnaire asks what makes it harder to consume the GDQS+ foods—fruits, vegetables, low-fat dairy, and deep orange tubers, and what makes it easier to consume the GDQS− foods—refined grains, sweets and ice cream, sugar-sweetened beverages, and red meat. Additionally, its inquiries about the frequency of whole grain consumption and the barriers if they are not consumed frequently. These questions cover a range of potential factors, including taste/texture preferences, cost, knowledge of health benefits, availability at home and in local markets, past eating habits, and spoilage rates (18–22).

### 2.2 Data processing

In addition to the multicomponent questionnaire, data collection involved the collection of 24-h recalls for the assessment of dietary intake. For each 24-HR, the interviewer obtained information regarding dietary intake during the past 24 h, related to the time of each meal’s intake, the food consumed by the subject, its portion size, preparation methods, and the brand of the food and beverages consumed, if applicable. For the estimation of portion size, common household measures used were measuring cups, spoons and plates. Nutritionist Pro software (version 8.1.0, 2023, Axial Systems) was used for the analysis of the dietary intake data and to estimate energy, and macro- and micronutrients’ intakes. For composite and mixed dishes, standardized recipes were added to the Nutritionist Pro software using single food items. Within the Nutritionist Pro, the USDA database was selected for analysis (SR 24, published September 2011).

In addition, food items, as consumed, were categorized into the 25 GDQS food groups. The quantity of each item consumed in grams per day was then classified into low, moderate, or high depending on the GDQS cut-off for each food group. Each food group for each participant was assigned a score, and the total GDQS score for each participant was calculated.

### 2.3 Statistical analysis

Descriptive statistics was presented as means and standard deviations (SD) for continuous variables and as frequencies (*n*) and proportions (%) for categorical variables.

GDQS score was calculated according to scores given to each food group, according to the quantity of consumption of each food group in grams/day. For 24 of the GDQS food groups, three ranges of quantity of consumption are defined (in grams/day) and used in scoring the metric: low, medium, and high. For one food group (high-fat dairy), four ranges of quantity of consumption are used: low, medium, high, and very high. The points associated with the healthy GDQS food groups increase for each higher quantity of consumption category. The points associated with the unhealthy GDQS food groups decrease for each higher quantity of consumption category. For the two food groups that are unhealthy in excessive consumption (red meat, high-fat dairy), the points associated with the GDQS food group increase up to a certain threshold of quantity of consumption, after which the points decrease (9).

The overall GDQS is a sum of the points across all 25 GDQS food groups. The GDQS has a possible range of 0 to 49. The GDQS+ is the total score across the 16 healthy GDQS food groups, with a possible range of 0 to 32. The GDQS− is the total score across the 7 unhealthy GDQS food groups and the 2 GDQS food groups that are unhealthy when consumed in excessive amounts, with a possible range of 0 to 17. A total GDQS score ≥ 23 is considered a “high” score which was shown to be associated with a low risk of nutrient inadequacy and NCD-related outcomes, GDQS ≥15 and < 23 is considered a “moderate” score which was shown to be associated with moderate risk, and GDQS <15 is considered a “low” score which was shown to be associated with high risk of nutrient inadequacy and NCD-related outcomes (9).

Due to the low percentage of subjects with high total GDQS scores, these were combined with the moderate total GDQS scores. Consequently, the total GDQS score levels were dichotomized into two categories: low total GDQS score and moderate-to-high total GDQS score. Frequencies and proportions were used to represent subjects with low, moderate, high, and very high intake for each GDQS food group among those with low total GDQS scores and those with moderate-to-high total GDQS scores. Differences between these two groups were examined using the chi-squared test and the two-sample z-test for proportions.

BMI was categorized into three groups: BMI <25, BMI 25–25.99 and BMI ≥30. Mean differences in total GDQS, GDQS+ and GDQS− scores between two or more groups were tested by independent samples *t*-test and one-way analysis of variance (ANOVA) with Bonferroni corrections, respectively. At the univariate level, association between GDQS score levels (low, moderate, and high) and socio-demographic, lifestyle and BMI characteristics were examined using chi-squared test. Binary logistic regression was then performed to account for potential confounders (which were identified based on the results of the univariate analyses, i.e., those variables that showed significant associations). The logistic regression analysis was conducted to analyze the relationship between the binary outcome variable (total GDQS score levels: low versus moderate-to-high) and various independent variables (sociodemographic, anthropometric and lifestyle characteristics). Odds Ratios (OR) and corresponding 95% confidence intervals (CI) were computed for each variable, with statistical significance set at *p* < 0.05. The Statistical Package for the Social Sciences (SPSS; version 25) was used for all computations and a *p*-value<0.05 was considered statistically significant.

For the drivers of consumption analysis, subjects with low intake of each of the selected GDQS healthy food groups and subjects with high intake of each of the selected GDQS unhealthy food groups were selected, and the frequencies and proportions for the drivers of consumption were presented only among these subjects.

Microsoft Excel (version; 16.67) was used to represent the drivers of consumption in bar charts.

## 3 Results

Table 1 displays the demographic, socioeconomic, anthropometric, and lifestyle characteristics of the study sample (*n* = 385 male students at AUB). The majority of participants were living at parental home (76.4%), in urban areas (85.5%), and pursued majors that were non-health related (76.6%). A large proportion of participants were categorized as non-drinkers or past drinkers (67.5%) and non-smokers or past smokers (74.8%). In terms of academic year, 29.4, 28.3, and 42.3% were in the first, second, and third year of study or beyond, respectively. Around 32% had a crowding index of more than one person per room, indicating lower socioeconomic status (SES) (23). More than half of the participants (64.7%) engaged in physical activity two or more times a week and 58% of the sample group performed 1–2 h of physical activity per day. Approximately 32.3% were overweight or obese and 2.3% were underweight.

**Table 1:** Socio-demographic, anthropometric and lifestyle characteristics in the sample of AUB male students.

Variable	Total (*n* = 385)
Demographic and socio-economic characteristics	
Age (years), mean (SD)	19.74 ± 1.52
Living arrangement, *n* (%)	
Living in parental home	294 (76.4)
Living in student residence	56 (14.5)
Living at their own home	35 (9.1)
Place of residence, *n* (%)	
Urban area	329 (85.5)
Rural area	56 (14.5)
Major of study, *n* (%)	
Health related major	90 (23.4)
Non-health related major	295 (76.6)
Academic year of study, *n* (%)	
First year university	113 (29.4)
Second year	109 (28.3)
≥ 3 years	163 (42.3)
Crowding index, *n* (%)	
< 1 person/room	250 (67.9)
≥ 1 person/room	118 (32.1)
Lifestyle characteristics	
Alcohol consumption status, *n* (%)	
Drinker	125 (32.5)
Non-drinker/past drinker	260 (67.5)
Smoking status, *n* (%)	
Current smoker	97 (25.2)
Non-smoker/past smoker	288 (74.8)
Physical activity frequency, *n* (%)	
Never or very rarely	25 (6.5)
Less than once a week	40 (10.4)
Once a week	71 (18.4)
Two or three times a week	113 (29.4)
More than three times a week	136 (35.3)
Physical activity duration per day	
Do not exercise	29 (7.5)
Less than 30 min	50 (13.0)
30 min	50 (13.0)
1–2 h	224 (58.2)
More than 2 h	32 (8.3)
Anthropometric characteristics	
Weight (Kg), mean (SD)	77.55 ± 13.0
Height (cm), mean (SD)	178.94 ± 6.42
Body mass index (BMI), mean (SD)	24.22 ± 3.85
BMI classifications, *n* (%)	
Underweight	9 (2.3)
Normal	251 (65.4)
Overweight	93 (24.2)
Obese	31 (8.1)
Overweight & obese	124 (32.3)

### 3.1 Evaluation of diet quality using GDQS and GDQS food groups consumption of the study sample

Table 2 displays the mean ± SD for total GDQS, GDQS+ and GDQS− scores alongside the percentages of subjects classified into low, moderate, and high total GDQS score categories. The mean values of total GDQS, GDQS+ and GDQS− scores were 15.4 ± 4.6, 6.0 ± 3.7, and 9.4 ± 2.8, respectively. The majority of the sample (95%) fell within the low and moderate GDQS categories with only 4.9% falling in the high GDQS category.

**Table 2:** Means of total GDQS, GDQS+, and GDQS− scores and the percentages of subjects with low, moderate, and high total GDQS score in the sample of AUB male students.

	Total (*n* = 385)
Total GDQS score, (mean ± SD)	15.4 ± 4.6
GDQS^+^ score, (mean ± SD)	6.0 ± 3.7
GDQS^−^ score, (mean ± SD)	9.4 ± 2.8
Total GDQS score levels, *n*, (%)	
Low (<15)	184 (47.8)
Moderate (15–23)	182 (47.3)
High (≥ 23)	19 (4.9)

GDQS+ Score: The total score across the 16 healthy GDQS food groups, with a possible range of 0 to 32. GDQS− Score: The total score across the 7 unhealthy GDQS food groups and the 2 GDQS food groups that are unhealth when consumed in excessive amounts, with a possible range of 0 to 17.

Table 3 illustrates the proportions of subjects in different intake categories (low, moderate, and high) for each food group based on GDQS score points.

**Table 3:** Percentages of subjects with low, moderate, high & very high intake category of each GDQS+ and GDQS− food groups in the sample of AUB male students.

GDQS food groups	Category of intake			
	Low	Moderate	High	Very high
	*n (%)*			
GDQS^+^ (Healthy)				
Citrus Fruits	320 (83.1)	15 (3.9)	50 (13)	-
Deep Orange Fruits	381 (99.0)	0 (0.0)	4 (1.0)	-
Other Fruits	217 (56.4)	17 (4.4)	151 (39.2)	-
Dark Green Leafy Vegetables	333 (86.5)	19 (4.9)	33 (8.6)	-
Cruciferous Vegetables	330 (85.7)	27 (7.0)	28 (7.3)	-
Deep Orange Vegetables	383 (99.5)	2 (0.5)	0 (0.0)	-
Other Vegetables	195 (50.6)	65 (16.9)	125 (32.5)	-
Legumes	317 (82.3)	8 (2.1)	60 (15.6)	-
Deep Orange Tubers	337 (87.5)	40 (10.4)	8 (2.1)	-
Nuts, Seeds	340 (88.3)	7 (1.8)	38 (9.9)	-
Whole Grains	333 (86.5)	3 (0.8)	49 (12.7)	-
Liquid Oils	143 (37.1)	21 (5.5)	221 (57.4)	-
Fish, Shellfish	377 (97.9)	1 (0.3)	7 (1.8)	-
Poultry Game Meat	158 (41.0)	6 (1.6)	221 (57.4)	-
Low Fat Dairy	376 (97.7)	0 (0.0)	9 (2.3)	-
Eggs	319 (82.9)	1 (0.3)	65 (16.9)	-
GDQS− (Unhealthy in excessive amounts)				
High Fat Dairy	232 (60.3)	69 (17.9)	77 (20.0)	7 (1.8)
Red Meat	247 (64.1)	40 (10.4)	98 (25.5)	-
GDQS− (Unhealthy)				
Processed meat	343 (89.1)	9 (2.3)	33 (8.6)	-
Refined Grains, Baked Goods	39 (10.1)	17 (4.4)	329 (85.5)	-
Sweets, Ice cream	205 (53.2)	22 (5.7)	158 (41.0)	-
Sugar Sweetened Beverages	234 (60.8)	19 (4.9)	132 (34.3)	-
Juice	326 (84.7)	5 (1.3)	54 (14.0)	-
White Roots Tubers	205 (53.2)	83 (21.6)	97 (25.2)	-
Purchased, Deep Fried Foods	227 (59.0)	31 (8.1)	127 (33.0)	-

GDQS+ food groups: The 16 healthy GDQS food groups. GDQS− food groups: The 7 unhealthy GDQS food groups and the 2 GDQS food groups that are unhealthy when consumed in excessive amounts. Low: The percentage of subjects consuming the food group at the lower end of its specific range of grams/day. Moderate: The percentage of subjects consuming the food group at a moderate level within its defined range of grams/day. High: The percentage of subjects consuming the food group at a higher level within its specified range of grams/day. Very high: The percentage of subjects consuming the food group at the highest level within its allotted range of grams/day.

For the GDQS+ food groups, the study sample exhibited low intake of citrus fruits (83.1%), deep orange fruits (99.0%) deep orange vegetables (99.5%), other fruits (56.4%), dark green leafy vegetables (86.5%), cruciferous vegetables (85.5%), deep orange vegetables (99.5%), other vegetables (50.6%), legumes (82.3%), deep orange tubers (87.5%), nuts and seeds (88.3%), whole grains (86.5%), fish and shellfish (97.9%), low fat dairy (97.7%), eggs (82.9%) along with a high intake of poultry and game meat (59.8%) and liquid oils (57.4%) among the GDQS+ groups.

Regarding GDQS− food groups, the study sample demonstrated a high intake of refined grains and baked goods. Conversely, there was a low intake observed for high fat dairy (60.3%) and red meat (64.1%).

### 3.2 Determination of the food groups contributing to a low GDQS score

Table 4 shows that individuals with low GDQS scores had significantly higher consumption of GDQS− food groups such as high-fat dairy, red meat, processed meat, sweets and ice-cream, sugar-sweetened beverages, white roots and tubers and purchased deep fried foods, compared to those with moderate/high GDQS scores. Conversely, individuals with moderate/high GDQS scores had significantly higher consumption of GDQS+ food groups such as citrus fruits, other fruits, dark green leafy vegetables, cruciferous vegetables, other vegetables, legumes, nuts and seeds, whole grains, liquid oils, poultry, low fat dairy products and eggs, compared to those with low GDQS scores.

**Table 4:** Comparison of the percentage of subjects with low, moderate, and high intake category of each food group between subjects with low and subjects with moderate/high total GDQS score.

Category of intake	Low total GDQS (*n* = 184)	Moderate/High total GDQS (*n* = 201)	Significance (*p*-value)
	*n* (%)		
GDQS^+^ (Healthy)			
Citrus fruits			**<0.001**
Low	174 (94.6) _a_	146 (72.6) _b_	
Moderate	1 (0.5) _a_	14 (7.0) _b_	
High	9 (4.9) _a_	41 (20.4) _b_	
Deep orange fruits			0.625
Low	183 (99.5) _a_	198 (98.5) _a_	
Moderate	0 (0.0)	0 (0.0)	
High	1 (0.5) _a_	3 (1.5) _a_	
Other Fruits			**<0.001**
Low	133 (72.3) _a_	84(41.8) _b_	
Moderate	10 (5.4) _a_	7 (3.5) _a_	
High	41 (22.3) _a_	110 (54.7) _b_	
Dark green leafy vegetables			**<0.001**
Low	178 (96.7) _a_	155 (77.1) _b_	
Moderate	4 (2.2) _a_	15 (7.5) _b_	
High	2 (1.1) _a_	31 (15.4) _b_	
Cruciferous vegetables			**0.011**
Low	167 (90.8) _a_	163 (81.1) _b_	
Moderate	6 (3.3) _a_	21 (10.4) _b_	
High	11 (6.0) _a_	17 (8.5) _a_	
Deep orange vegetables			0.950
Low	183 (99.5) _a_	200 (99.5) _a_	
Moderate	1 (0.5) _a_	1 (0.5) _a_	
High	0 (0.0)	0 (0.0)	
Other vegetables			**<0.001**
Low	123 (66.8) _a_	72 (35.8) _b_	
Moderate	20 (10.9) _a_	45 (22.4) _b_	
High	41 (22.3) _a_	84 (41.8) _b_	
Legumes			**<0.001**
Low	178 (96.7) _a_	139 (69.2) _b_	
Moderate	0 (0)_a_	8 (4.0) _b_	
High	6(3.3) _a_	54 (26.9) _b_	
Deep orange tubers			0.046
Low	169 (91.8) _a_	168 (83.6) _b_	
Moderate	13 (7.1) _a_	27 (13.4) _b_	
High	2 (1.1) _a_	6 (3.0) _b_	
Nuts, seeds			**<0.001**
Low	177 (96.2) _a_	163 (81.1) _b_	
Moderate	3 (1.6) _a_	4 (2.0) _a_	
High	4 (2.2) _a_	34 (16.9) _b_	
Whole grains			**<0.001**
Low	175 (95.1) _a_	158 (78.8) _b_	
Moderate	0 (0.0) _a_	3 (1.5) _a_	
High	9 (4.9) _a_	40 (19.9) _b_	
Liquid oils			**<0.001**
Low	101 (54.9) ^a^	42 (20.9) ^b^	
Moderate	6 (3.3) ^a^	15 (7.5) ^a^	
High	77 (41.8) ^a^	144 (71.6) ^b^	
Fish, shellfish			0.560
Low	180 (97.8) _a_	197 (98.0) _a_	
Moderate	0 (0.0) _a_	1 (0.5) _a_	
High	4 (2.2) _a_	3 (1.5) _a_	
Poultry game meat			**0.026**
Low	83 (45.1) _a_	75 (37.3) _a_	
Moderate	0(0.0) _a_	6 (3.0) _b_	
High	101(54.9) _a_	120 (59.7) _a_	
Low fat dairy			**0.004**
Low	184(100) _a_	192 (95.5) _a_	
Moderate	0 (0.0)	0 (0.0)	
High	0 (0.0) _a_	9 (4.5) _b_	
Eggs			**0.028**
Low	162 (88.0) _a_	157(78.1) _b_	
Moderate	0 (0.0) _a_	1 (0.5) _a_	
High	22 (12.0) _a_	43 (21.4) _b_	
GDQS^−^ (Unhealthy in excessive amounts)			
High fat dairy			**0.001**
Low	125 (67.9) _a_	107 (53.2) _b_	
Moderate	35 (19.0) _a_	34 (16.9) _a_	
High	21 (11.4) _a_	56 (27.9) _b_	
Very high	3 (1.6) _a_	4 (2.0) _a_	
Red meat			**<0.001**
Low	129 (70.1) _a_	118 (58.7) _b_	
Moderate	7 (3.8) _a_	33(16.4) _b_	
High	48 (26.1) _a_	50 (24.9) _a_	
GDQS− (Unhealthy)			
Processed meat			**0.005**
Low	154 (83.7) _a_	189(94.0) _b_	
Moderate	6 (3.3) _a_	3 (1.5) _a_	
High	24 (13.0) _a_	9 (4.5) _b_	
Refined grains, baked goods			0.899
Low	20 (10.9) _a_	19 (9.5) _a_	
Moderate	8 (4.3) _a_	9 (4.5) _a_	
High	156 (84.8) _a_	173 (86.1) _a_	
Sweets, ice cream			**<0.001**
Low	70 (42.9) _a_	126 (62.7) _b_	
Moderate	6 (3.3) _a_	16 (8.0) _b_	
High	99 (53.81) _a_	59 (29.4) _b_	
Sugar sweetened beverages			**<0.001**
Low	88 (47.8) _a_	146 (72.6) _b_	
Moderate	7 (3.8) _a_	12 (6.0) _a_	
High	89 (48.4) _a_	43 (21.4) _b_	
Juice			0.055
Low	150 (81.5) _a_	176 (87.6) _a_	
Moderate	1 (0.5) _a_	4 (2.0) _a_	
High	33 (17.9) _a_	21 (10.4) _b_	
White roots tubers			**<0.001**
Low	71 (38.6) ^a^	134 (66.7) ^b^	
Moderate	46 (25.0) ^a^	37 (18.4) ^a^	
High	66 (36.1) ^a^	30 (14.9) ^b^	
Purchased, deep fried foods			**<0.001**
Low	82 (44.6)_a_	145 (72.1)_b_	
Moderate	13 (7.1)_a_	18 (9.0)_a_	
High	89 (48.4)_a_	38 (18.9)_b_	

Numbers in bold face indicate statistical significance (*p*-value < 0.05). ^a, b^ Values of different subscripts are statistically significant at *p* < 0.05 using comparison of column proportions (z-test) for categorical variables. Low total GDQS: Percentage of subjects with a total GDQS score < 15. Moderate/high total GDQS: Percentage of subjects with a total GDQS score ≥ 15.

Table 5 presents the association between sociodemographic, anthropometric and lifestyle characteristic with GDQS scores, including GDQS, GDQS+ and GDQS− scores. Significant variations in GDQS scores were noted across different living arrangements, academic year of study and majors of study. Specifically, living arrangement was significantly associated with GDQS− scores with students living alone exhibiting notably higher GDQS− scores compared to those residing with their parents or in student residences. Regarding majors of study, there was a significant association between GDQS+ scores and students enrolled in health-related fields. In terms of academic year of study, significantly differences were observed in total GDQS and GDQS+ scores between first year students and students in their second year of beyond, with second-year students achieving have the highest scores in these categories.

**Table 5:** Mean GDQS, GDQS+ and GDQS− scores according to socio-demographic, lifestyle, and BMI characteristics in the sample of AUB male students.

Variables	Total GDQS mean ± SD	*p*-value^*^	GDQS^+^ mean ± SD	*p*-value^*^	GDQS^−^ mean ± SD	*p*-value^*^
Living arrangement		0.090		0.782		**0.023**
Living at parental home	15.35 ± 4.66		6.04 ± 3.75		9.32 ± 2.69	
Living at student residence	14.75 ± 4.44		5.82 ± 3.13		8.93 ± 3.27	
Living at their own home	16.89 ± 4.08		6.37 ± 3.72		10.51 ± 2.33	
Place of residence		0.600		0.607		0.849
Urban area	15.46 ± 4.71		6.07 ± 3.68		9.38 ± 2.82	
Rural area	15.11 ± 3.90		5.80 ± 3.59		9.30 ± 2.43	
Major of study		0.056		**0.014**		0.959
Health related major	16.221 ± 4.35		6.86 ± 3.57		9.36 ± 2.79	
Non-health related major	15.16 ± 4.65		5.78 ± 3.66		9.37 ± 2.77	
Academic year of study		**<0.001**		**<0.001**		0.201
First year university	14.04 ± 4.22		4.95 ± 3.34		9.09 ± 2.84	
≥2 years	15.97 ± 4.63		6.48 ± 3.69		9.48 ± 2.73	
Crowding index		0.796		0.802		0.923
<1 person/room	15.53 ± 4.58		6.11 ± 3.70		9.42 ± 2.79	
≥1 person/room	15.40 ± 4.65		6.01 ± 3.70		9.40 ± 2.67	
Alcohol consumption status		0.202		0.577		0.168
Drinker	14.97 ± 4.78		5.89 ± 3.86		9.09 ± 2.77	
Non-drinker/past drinker	15.61 ± 4.50		6.11 ± 3.56		9.50 ± 2.77	
Smoking status		0.424		0.671		0.427
Current smoker	15.07 ± 4.70		5.90 ± 3.63		9.18 ± 2.96	
Non-smoker/past smoker	15.52 ± 4.56		6.08 ± 3.67		9.43 ± 2.70	
Physical activity frequency		0.103		0.080		0.696
<2 times/week	14.56 ± 4.63		5.31 ± 3.53		9.25 ± 2.83	
≥2 times/week	15.58 ± 4.58		6.18 ± 3.67		9.39 ± 2.76	
Physical activity duration		0.828		0.775		0.987
<1 h/day	15.33 ± 4.62		5.96 ± 3.71		9.37 ± 2.92	
≥1 h/day	15.44 ± 4.59		6.07 ± 3.64		9.37 ± 2.70	
BMI		0.528		0.682		0.762
<25	15.56 ± 4.67		6.13 ± 3.62		9.42 ± 2.78	
25–29.9	15.28 ± 4.34		5.92 ± 3.58		9.35 ± 2.80	
≥30	14.59 ± 4.82		5.56 ± 4.27		9.03 ± 2.71	

^*^p-value is derived from independent samples *t*-test and ANOVA for all continuous variables. Numbers in bold face are statistically significant (*p*-value < 0.05). GDQS Score: The total score across the 25 GDQS food groups with a possible range of 0 to 49. GDQS+ Score: The total score across the 16 healthy GDQS food groups, with a possible range of 0 to 32. GDQS− Score: The total score across the 7 unhealthy GDQS food groups and the 2 GDQS food groups that are unhealth when consumed in excessive amounts, with a possible range of 0 to 17.

Table 6 presents the results of a binary logistic regression analysis examining the association between sociodemographic, lifestyle factors, BMI characteristics, and low GDQS score (<15) among AUB male students, after adjusting for confounders (which were identified based on the significant univariate associations shown in Table 5 and which included living arrangements; major of study; academic year of study). Significant associations were found between low GDQS score and major of study (non-health related major), academic year of study (second year and beyond), and BMI classification (obese ≥30 kg/m^2^). More specifically, individuals with obesity exhibit a 57% lower likelihood of achieving a high GDQS score (OR: 0.43; 95% CI: 0.19–0.97). Those in non-health related majors have a 44% lower likelihood of achieving a high GDQS score compared to those in health-related majors (OR: 0.56, 95% CI: 0.33–0.94). Conversely, students in their second academic year demonstrate nearly two folds higher likelihood of having a high GDQS score compared to those in their first year (OR: 1.94; 95% CI: 1.19–3.15).

**Table 6:** Association of sociodemographic, lifestyle and BMI characteristics with low GDQS score (<15) among AUB male students using binary logistic regression.

Variable	Low GDQS < 15	
	OR (95 %CI)	*p*-Value
Living arrangement		
Living at parental home	1	
Living at student residence	0.79 (0.42–1.52)	0.482
Living at their own home	1.33 (0.62–2.85)	0.466
Place of residence		
Urban area	1	
Rural area	0.76 (0.42–1.39)	0.375
Major of study		
Health related major	1	
Non-health related major	0.56 (0.33–0.94)	**0.029**
Academic year of study		
First year university	1	
≥2 years	1.94 (1.19–3.15)	**0.007**
Crowding index		
<1 person/room	1	
≥ 1 person/room	1.08 (0.68–1.71)	0.744
Alcohol consumption status		
Drinker		
Non-drinker/past drinker	1.43 (0.88–2.33)	0.154
Smoking status		
Current smoker	1	
Non-smoker/past smoker	0.88 (0.53–1.48)	0.634
Physical activity frequency		
< 2 times/week	1	
≥ 2 times/week	1.59 (0.79–3.23)	0.192
Physical activity duration		
< 1 h/day	1	
≥ 1 h/day	0.84 (0.47–1.47)	0.538
BMI Classification		
Normal<25 kg/m^2^	1	
Overweight 25–29.9	0.99 (0.59–1.66)	0.991
Obese≥30	0.43 (0.19–0.97)	**0.043**

Numbers in bold face are statistically significant (*p*-value < 0.05).

Sensitivity analyses were conducted where the covariates selected for the binary logistic regression model were chosen based on their observed associations in univariate analyses and their theoretical role as potential confounders in the relationship between GDQS scores and the outcome variables (9–15). This approach was informed by both the data-driven results and existing literature. Therefore, the analyses were adjusted for living arrangement, place of residence, major of study, academic year of study, crowding index, alcohol consumption status, smoking status, physical activity frequency, physical activity duration and BMI classification. A test for multicollinearity was conducted in the regression analysis, and there was no evidence for collinearity between the independent variables. The sensitivity analysis yielded similar findings as those shown in Table 6. More specifically, individuals with obesity exhibited a 56% lower likelihood of achieving a high GDQS score (OR: 0.44; 95% CI: 0.20–0.97).

### 3.3 Drivers of eating behavior

**Figure 1:**
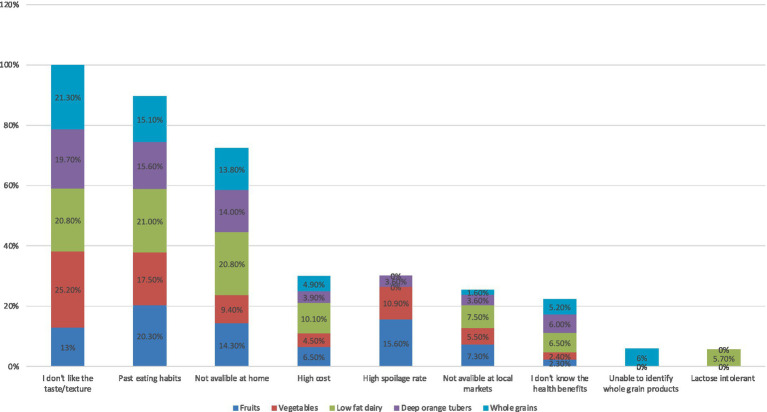
Perceived barriers to consumption of healthy food groups.

Figure 1 outlines perceived barriers to the consumption of GDQS+ food groups including fruits, vegetables, low fat dairy, deep orange tubers and whole grains. Among the reported barriers, taste taste/texture dislike, past eating habits, unavailability at home emerged as the most common barriers, with taste dislike being the highest for vegetables, while past eating habits and unavailability at home were higher barriers for low-fat dairy. Difficulty in identifying products was uniquely reported as a barrier for whole grains consumption while lactose intolerance was specifically mentioned as an obstacle to consuming low-fat dairy products.

**Figure 2:**
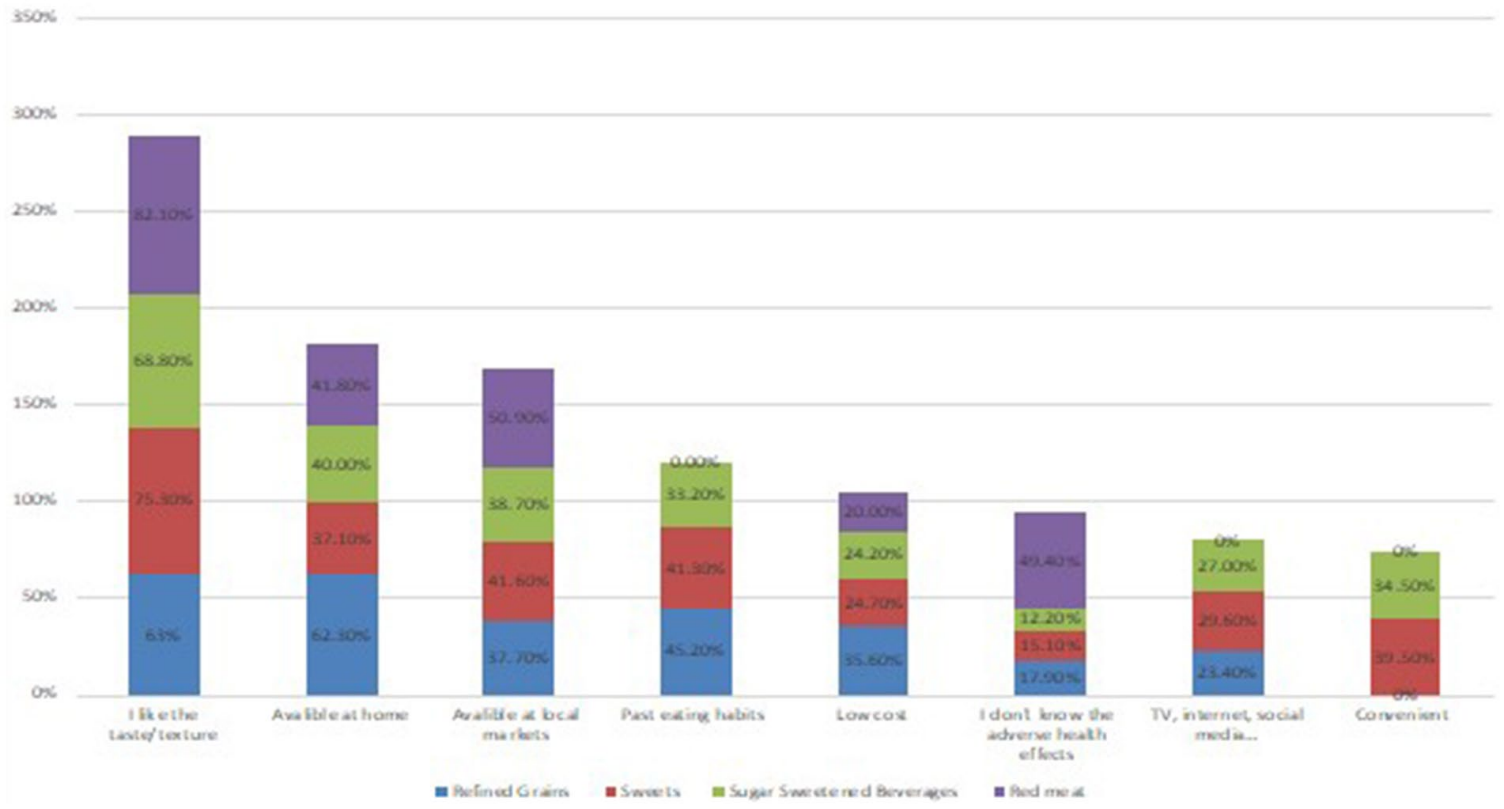
Perceived facilitators to consumption of unhealthy food groups.

Figure 2 presents the perceived facilitators to the consumption of GDQS-unhealthy food groups including refined grains, sweets and ice-cream, sugar sweetened beverages and red meat. Among the reported facilitators, liking taste/texture emerged as the most commonly reported factor followed by similar trends for availability at home and availability at local markets, with taste/texture preference and availability at local markets being the highest for red meat, while availability at home was highest for refined grains.

## 4 Discussion

This is the first study to use the GDQS metric to assess diet quality in relation to NCDs’ risk and nutrient adequacy among male university students in Lebanon.

The findings of this study show that a substantial proportion of the sample exhibited low GDQS scores, indicative of an elevated risk for NCDs. This is of concern given NCDs are responsible for a staggering 91% of all deaths in Lebanon (2) with CVDs standing out as the primary contributor, accounting for 47% of all-cause mortality (3).

When comparing the GDQS results obtained in this study with those from other populations, a notable trend emerges with countries falling within the low to middle income bracket typically exhibiting GDQS scores ranging from low to moderate, while high-income countries tend to have higher GDQS scores. For instance, the average GDQS score of 15.4 ± 4.6 in our study surpassed that of Mexico (13) and Brazil (24) which recorded average GDQS scores of 6.4 ± 4.0 amongst nonpregnant nonlactating Mexican women of reproductive age, and 14.5 ± 0.04 amongst men and non-pregnant and non-lactating women in Brazil, respectively. However, the score estimated in our study fell below that of China (10), India (11), Sub-Saharan Africa (12) and Vietnam (25) with respective average GDQS scores of 19.8, 23, 22.5 and 17.9 amongst men and women aged >18 y, nonpregnant women of reproductive age, men and nonpregnant nonlactating women of reproductive age (15–49 y), and young women aged 16–22 years, respectively. Despite these variations, all the aforementioned low- to middle-income countries consistently scored within the low to moderate range of the score, indicating a heightened risk for NCDs. Conversely, Lebanon average GDQS score was lower than that of Greece (26) and the United States (14), both categorized as high-income countries, with respective average GDQS scores of 27 ± 14 in adult men and women and 28.8 ± 2.2 in women aged 27–44 y, respectively, a fact that indicate better quality diets in these countries and hence a lower risk for NCDs.

Studying the consumption of food groups that contributed to the low GDQS scores revealed high intake of foods such as processed meat, refined grains, sweets and ice cream, sugar-sweetened beverages, juices, white roots and tubers, and purchased deep fried foods. Research has shown that these foods increase the risk for NCDs through their cardiometabolic effects. For instance, processed meats have been linked to type 2 diabetes and cardiovascular disease, including coronary heart disease. Preservatives in processed meats, such as nitrates and nitrites, are associated with endothelial dysfunction and impaired insulin responses, and consumption of processed meats increase the risk of hypercholesterolemia, hypertriglyceridemia, and dyslipidemia (27). Refined carbohydrates can cause significant fluctuations in plasma glucose and insulin levels, as well as adiposity and insulin resistance (28). Excessive sugar consumption is linked to obesity, cardiovascular diseases, type 2 diabetes mellitus, metabolic syndrome, and nonalcoholic fatty liver disease. Obesity, which is a primary risk factor for sleep-disordered breathing, is linked to higher body weight, adiposity, decreased insulin sensitivity, hyperglycemia, and cardiometabolic risk factors (29). Furthermore, sugar-sweetened beverages are linked to weight gain and heightened risks of type 2 diabetes mellitus, CVDs, and certain cancers. They induce hyperinsulinemia from rapid glucose absorption and contribute to chronic disease risk through adverse glycemic effects and hepatic metabolism of excess fructose (30). Epidemiological studies have linked potato intake with obesity, type 2 diabetes due to its high glycemic index and cardiovascular disease (31). Fried-food consumption may influence blood pressure and frying produces a considerable amount of trans-fatty acids that elevate LDL-cholesterol and reduce HDL-cholesterol levels (32).

Additionally, research into food groups contributing to the low score also revealed a low intake of GDQS+ food groups such as fruits (citrus, deep orange, other fruits), vegetables (deep orange, dark green leafy, cruciferous, other vegetables), legumes, deep orange tubers, nuts and seeds, whole grains, fish and shellfish, low fat dairy and eggs. Studies have shown that consuming these foods is crucial for mitigating the risk of NCDs. For instance Citrus fruits are recognized for their anti-oxidative, anti-inflammatory, and cardiovascular protective effects (33). Cruciferous vegetables, rich in beneficial compounds such as carotenoids, flavonoids, anthocyanins and antioxidants (34) are linked to a reduced risk of cardiometabolic diseases, musculoskeletal conditions, and cancer (35). Green leafy vegetables which are important sources of Lutein, iron and vitamin A are associated with a decreased risk of all-cause mortality, coronary heart disease and stroke (36, 37). Legumes have been shown to lower the risk of type 2 diabetes, improve glycemic control for those with diabetes, reduce total and LDL cholesterol levels and improve blood pressure (38). Consumption of nuts and seeds and whole grains was also shown to be inversely associated with cardiovascular disease (39), diabetes and various types of cancer (40). Liquid oils, such as virgin olive oil, are associated with reduced cardiovascular risk, lower body mass index, improved blood pressure and anti-inflammatory effects (41). Additionally, eggs can decrease appetite and have protective effects against certain cancers and hypertension (42).

Taken together, the low GDQS scores observed in the present study are primarily attributed to the low consumption of fruits (citrus fruits and other fruits), vegetables (dark green leafy vegetables, cruciferous vegetables and other vegetables) legumes, nuts and seeds, whole grains, liquid oils, poultry, low fat dairy products and eggs and the high consumption of processed meat, sweets and ice-cream, sugar-sweetened beverages, white roots and tubers and purchased deep fried foods. These findings align with previous studies conducted on different populations using the GDQS. The low consumption of fruits and vegetables was observed in Brazil (24), China, (notably low in consumption of deep orange fruits, citrus fruits, other fruits, cruciferous vegetables and deep orange vegetables) (10), India, (low in consumption of citrus fruits, deep orange fruits, cruciferous vegetables and deep orange vegetables) (11), and Vietnam (low consumption of deep orange fruits and dark green leafy vegetables) (25). Additionally, a common trend observed across all 4 populations is the low consumption of whole grains and the high intake of refined grains and baked goods (10, 11, 24, 25).

In this study, it was demonstrated that students majoring in health-related disciplines exhibited higher GDQS scores than those majoring in non-health related disciplines. This suggests that individuals in health-related majors may possess more knowledge about nutrition and health, and that their academic training and exposure to health-related information likely contributed to their better adherence to dietary guidelines and healthier lifestyle choices. A study conducted to assess nutrition knowledge, attitudes, and lifestyle practices (KAP) related to breast cancer risk reduction among female university students in Lebanon also showed that pursuing a health-related major as well as a higher GPA were linked to better knowledge and attitudes, and improved nutrition-related knowledge (43). These findings also align with data from the UK, where university students enrolled in healthcare disciplines demonstrated higher median scores in nutrition knowledge compared to those in non-healthcare disciplines (44). This suggests that education in health-related disciplines plays a crucial role in promoting healthier dietary habits and lifestyles.

A notable finding in this study was the link between low GDQS scores and obesity in the study sample. Such findings align with previous research conducted amongst university students, where poor dietary habits were linked to increased obesity risk. For instance, a study conducted on university students in Chile (45) highlighted the association between obesity and the consumption of ultra-processed foods such as sugary beverages. Additionally, a study on university students in Bangladesh (46) revealed that consuming four or more meals a day, along with frequent intake of junk food, fast food, and soft drinks (three or more days a week), were potential determinants of overweight and obesity in this population. Furthermore, a study done on university students in Saudi Arabia (47) found a higher prevalence of obesity among male students with increased consumption of sugary beverages, smoking, and inadequate sleep, which emerged as independent predictors of obesity in the study population.

In this study, it was observed that university students in their second year and beyond displayed higher GDQS scores than those in their first year. This discrepancy may be attributed to the greater health literacy associated with higher educational level and exposure, compared to lower ones. In a study conducted on university students in Turkey (48), results indicated that the health literacy of freshman university students in various education programs was at a medium level and as education level increased, health literacy also increased. Studies also conducted on university students in Jordan (49) and nursing students in Turkey (50) reported that students in higher years of study have a better health literacy level compared with those in the first year of study. One possible reason for this could be the availability of various educational resources aimed towards health promotion throughout the later years of university study such as seminars, awareness campaigns, health-related elective courses, and additional educational initiatives.

A significant association was observed between GDQS− scores and living arrangements, with those living in student residences exhibiting lower GDQS− scores in this study. This pattern aligns with findings from reported in the literature. A study conducted amongst university students in Greece (51) revealed that students living away from the family home decreased their weekly consumption of fresh fruit, cooked and raw vegetables, oily fish, seafood, pulses and olive oil, and increased their sugar, wine, alcohol and fast-food intake. This is in line with findings from a study conducted among undergraduate students in Italy (52) where students living away from home consumed more packaged/ready food, beer and spirits, milk and chips, and reported a modification of dietary habits since leaving family.

Studying the barriers to consuming of GDQS+ food groups in our study revealed that taste was the most significant factor followed by past eating habits, unavailability at home, high cost, high spoilage rate, unavailability at local markets, lack of knowledge about the health benefits, difficulty identifying products and lactose intolerance. On the other hand, examining the facilitators for consuming of GDQS− food groups showed that factors, such liking the taste/texture, availability at home, followed by availability at local markets, past eating habits, low cost, lack of awareness of adverse health effects, TV, internet and social media and convenience played a significant role. These findings are consistent with those reported from other countries. For instance, in Kuwait, taste, inconvenience, and lack of knowledge about fruits and vegetable intake recommendations and preparation methods were major barriers to consuming more fruits and vegetables among university students (53). Similarly, data from college students in the US indicated that common barriers to healthy eating included time constraints, unhealthy snacking, convenience high-calorie food, stress, high prices of healthy food, and easy access to junk food (54). In KSA, a study conducted among King Faisal University students found that barriers to adhering to healthy eating included the availability of fast food, the high cost of healthy food, limited time, and laziness (55). Regarding whole grains, a study among Iranian students identified obstacles such as access issues, family supply issues, lack of appeal, non-consumption by classmates, and recent increases in prices (56).

Although this study focused on barriers to consuming specific food groups, it is important to recognize that various other factors beyond the study’s scope may have influenced food consumption patterns. While cost did not appear as a determining factor in this study, the economic crises in Lebanon likely exacerbated these issues, impacting food security and accessibility (57). Low consumption of legumes may stem from taste preferences, digestive discomfort, or concerns about carbohydrate content (58). Additionally, confusion about the effects of nuts on weight, their cost, dental concerns, and allergies may have contributed to their low intake (59). Factors such as lack of access, attractiveness, price, parental influence, peer pressure, and lack of nutrition label awareness could all affect whole grain consumption (56). Misconceptions about cholesterol levels in eggs and their association with CVD and cancer mortality could contribute to reduced egg consumption, despite evidence suggesting their health benefits (60).

Further analysis of GDQS scores from a previous national study conducted in 2008/09 on a nationally representative sample of Lebanese adults, utilizing 24-h recall (24HR) data (61), revealed average GDQS scores similar to the current study (data not shown). However, notable changes in the consumption of the GDQS+ food groups and GDQS− food groups were observed. For the GDQS+ food groups, there was a lower consumption of citrus fruits, other fruits, other vegetables, legumes, and poultry and game meat, but a higher consumption of dark green leafy vegetables, deep orange tubers, nuts and seeds, liquid oils, and fish and shellfish in the present study compared to 2008/09 data. As for the GDQS− food groups, there was a higher consumption of juice and purchased deep-fried foods, alongside a lower consumption of refined grains and baked goods, sweets and ice cream, and sugar-sweetened beverages in the present study compared to 2008/09. These findings emphasize the importance of continuous monitoring of food intake over time.

This study has several strengths. It is the first time that the GDQS score is applied in a Middle Eastern population. However, this study has certain limitations as it was conducted on a small sample and its findings cannot be generalized to the whole population as it was restricted to AUB male students only who, in general, belong to a higher socio-economic status. Further studies are therefore needed to investigate GDQS on a larger and more representative sample of the Lebanese population. In addition, the cross-sectional design of the study does not allow to infer causality but rather serves to show associations without temporal sequence. Moreover, a single day’s intake of food is not a strict measure of the overall food consumption practices, however, this method is consistent with the GDQS protocol applied in other countries and is useful for assessing dietary habits at a population or group level. Other limitations may pertain to the GDQS itself, the validity of its associations with NCDs in different populations and the categorization of foods into GDQS^+^ and GDQS^−^. For instance, it has been argued that different scoring methods may yield different associations between food groups and NCDs, in a way that the same food group can get associated with either beneficial or harmful effects on NCD risk (62). Additionally, although this study findings suggest an association between the GDQS score and obesity as a non-communicable disease (NCD), it is important to recognize that the score itself does not consider other lifestyle factors, such as physical activity levels, smoking status, alcohol consumption and overall health status, which may co-occur with the consumptions of GDQS− foods and which can explain at least some of the associations (63).

This study showed that the Global Diet Quality Score (GDQS) as a valuable tool for assessing the dietary habits of young adults in Lebanon, an observation supported not only by this study but also by the findings of studies conducted in diverse populations worldwide. The study findings showed an alarmingly high percentage of young Lebanese male students with low GDQS score, alongside a very small percentage with high GDQS score, thus indicating a high risk for NCDs.

It also identified the specific foods whose consumption should be further increased or decreased in the study population and identified some of the barriers to consumption of favorable foods. Findings of this study call for larger investigations of diet quality to provide much needed evidence for the development of context-specific interventions aimed at encouraging healthier eating habits among Lebanese university students to help combat the rise of NCDs in the country and emphasize the importance of implementing strategies that encompass increased health-related education, and enhanced access to healthy food options on campus.

## Data Availability

The original contributions presented in the study are included in the article/supplementary material, further inquiries can be directed to the corresponding authors.
